# Tuning the photo-response in monolayer MoS_2_ by plasmonic nano-antenna

**DOI:** 10.1038/srep23626

**Published:** 2016-03-31

**Authors:** Jiu Li, Qingqing Ji, Saisai Chu, Yanfeng Zhang, Yan Li, Qihuang Gong, Kaihui Liu, Kebin Shi

**Affiliations:** 1 State Key Laboratory for Mesoscopic Physics, Collaborative Innovation Center of Quantum Matter, School of Physics, Peking University, Beijing 100871, China; 2 Collaborative Innovation Center of Extreme Optics, Shanxi University, Taiyuan, Shanxi 030006, China; 3 Center for Nanochemistry (CNC), Beijing National Laboratory for Molecular Sciences, College of Chemistry and Molecular Engineering, Academy for Advanced Interdisciplinary Studies, Peking University, Beijing 100871, People’s Republic of China

## Abstract

Monolayer molybdenum disulfide (MoS_2_) has recently attracted intense interests due to its remarkable optical properties of valley-selected optical response, strong nonlinear wave mixing and photocurrent/photovoltaic generation and many corresponding potential applications. However, the nature of atomic-thin thickness of monolayer MoS_2_ leads to inefficient light-matter interactions and thereby hinders its optoelectronic applications. Here we report on the enhanced and controllable photo-response in MoS_2_ by utilizing surface plasmonic resonance based on metallic nano-antenna with characteristic lateral size of 40 × 80 nm. Our nano-antenna is designed to have one plasmonic resonance in the visible range and can enhance the MoS_2_ photoluminescence intensity up to 10 folds. The intensity enhancement can be effectively tuned simply by the manipulation of incident light polarization. In addition, we can also control the oscillator strength ratio between exciton and trion states by controlling polarization dependent hot carrier doping in MoS_2_. Our results demonstrate the possibility in controlling the photo-response in broad two-dimensional materials by well-designed nano-antenna and facilitate its coming optoelectronic applications.

As a new attractive material after graphene in the family of two-dimensional materials, monolayer MoS_2_ has drawn intense interests due to its intriguing physical properties, including a direct optical band gap in the visible range^1^,^2^, a strong exciton binding energy^3^,^4^, and valley selective circular dichroism MoS_2_^5^,^6^,^7^. Monolayer MoS_2_, together with its sibling materials of WS_2_, MoSe_2_, WSe_2_, MoTe_2_, WTe_2_, have shown great potential in the applications of nano-electronics^8^, photonics^9^,^10^, photovoltaics^11^,^12^, and valleytronics^13^,^14^. However, as an optoelectronic material, the light-matter interaction cross-section of monolayer MoS_2_ is still too small for practical device applications due to its atomic-thin characteristic. Therefore, it is highly desirable to enhance the light-matter interaction strength in monolayer MoS_2_. Many efforts have been made to achieve this goal^15^,^16^,^17^,^18^, and surface plasmonic resonance (SPR) turns out to be very attractive due to its simplicity and high-efficiency^19^,^20^. It has been demonstrated that the metallic nanoparticles can enhance the photocurrent in MoS_2_ field-effect transistors^21^,^22^ and also the light emission^23^; 100 × 1000 nm-sized metal nano-antenna can improve the photo-response to some extent^24^. Till now, the significant enhancement of optical response in a well-controlled scheme has not been realized yet.

Here we report on the greatly enhanced and polarization controllable photoluminescence (PL) in monolayer MoS_2_ by fabricating metallic nano-antenna onto MoS_2_ surface. The large photo-response enhancement is realized by designing the periodic nano-antenna with characteristic lateral size of sub-100 nm to produce a plasmonic resonance matching with the excitation laser energy; the polarization controllability comes from the asymmetric optical field enhancement at the direction parallel and perpendicular to the long axis of nano-antenna. We readily realized up to one order of magnitude enhancement of PL and this enhancement can be well-controlled by incident light polarization. In addition, we show that the shifting of the PL peak positions can be achieved by controlling the oscillator strength ratio between exciton and trion states using polarization manipulation. Our results demonstrate the possibility in controlling the photo-response in broad two-dimensional materials by well-designed nano-antenna structures and facilitate its coming potential applications in optoelectronics and photovoltaics.

## Results

**Figure 1:**
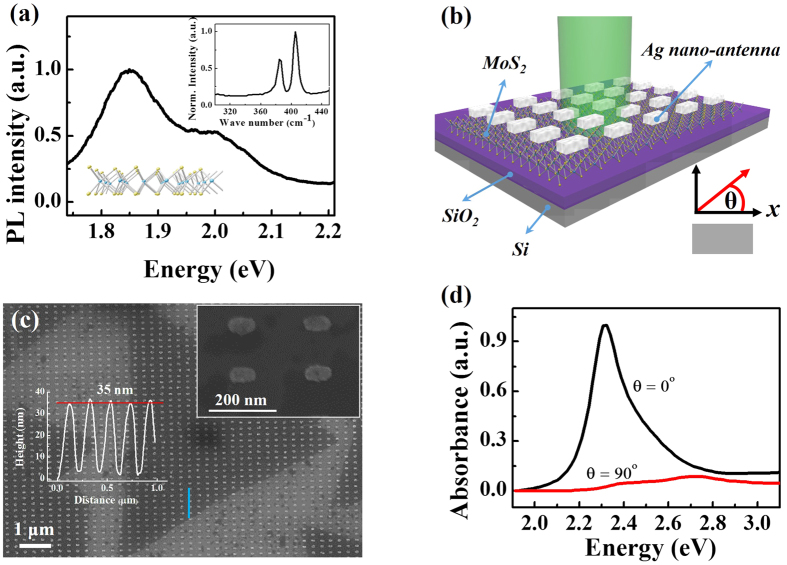
(**a**) The PL spectrum of pristine monolayer MoS_2_, displaying two fingerprinting peaks at ~ 1.85 eV and 2.00 eV. Inset: the Raman spectrum of pristine monolayer MoS_2_. The observed in-plane mode 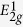 and out-of-plane mode *A*_1*g*_ were at 381 cm^−1^ and 400 cm^−1^, respectively. (**b**) Scheme of periodic Ag nano-antenna arrays on monolayer MoS_2_. (**c**) The SEM image of nano-antenna arrays. The in-plane size of nano-antenna is ~ 40 × 80 nm (inset upper right). Its height is measured at ~ 35 nm by using AFM scanning along the marked blue line, the result is shown as inset (left). (**d**) The simulated absorbance spectrum of single nano-antenna with polarization parallel (θ = 0°) and vertical (θ = 90°) to the long axis of nano-antenna.

In our experiment, monolayer MoS_2_ was controllably synthesized on mica through a low-pressure chemical vapor deposition process^25^ and then transferred onto SiO_2_/Si substrate. PL (Fig. 1a) and Raman spectra (inset) confirmed the monolayer nature of our MoS_2_ sample: the PL spectrum shows two peaks related to A exciton at ~ 1.85 eV and B exciton at ~ 2.00 eV, and the *A*_1*g*_ and 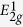 Raman modes are separated by about^26^ 19 cm^−1^. Since the absorbance spectrum of MoS_2_ is in the visible range, nanostructures with visible wavelength SPR are primarily considered in order to match the absorbance of MoS_2_ efficiently. Here we employed standard electron beam lithography and pulse laser deposition to fabricate silver nano-antenna patterns on monolayer MoS_2_ (Fig. 1b). The characteristic lateral size of single nano-antenna structure is ~ 40 × 80 nm with thickness of ~ 35 nm (Fig. 1c). The incident laser polarization has an θ angle relative to the long axis of nano-antennas. To have large photo-response, our nano-antennas are intentionally designed to have one resonance matching our excitation laser of 2.34 eV (532 nm). Our simulation results in Fig. 1d showed that in the 2.0–3.0 eV spectral region our nano-antennas have strong longitudinal mode (θ = 0°) absorption with peak at ~ 2.34 eV but negligible transverse mode (θ = 90°) absorption. Therefore, we can purposely choose the 532 nm linear-polarized incident laser to efficiently excite the longitudinal mode of nano-antenna SPR without exciting the transverse mode too much.

**Figure 2:**
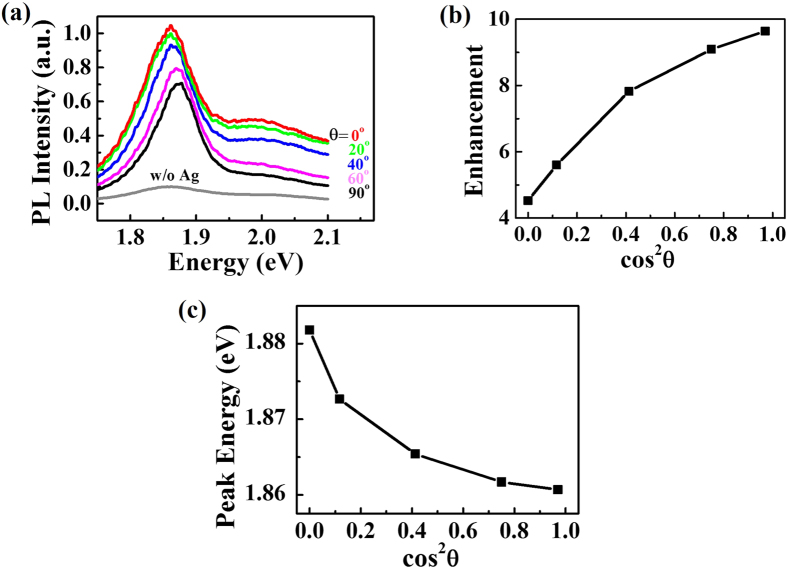
Polarization dependent PL intensity and peak position. (**a**) PL spectra in Ag nano-antenna coated MoS_2_ under excitation with different polarization angles θ, comparing with pristine monolayer MoS_2_. Maximum of 10 times PL enhancement was observed. (**b**) The dependence of integrated PL peak intensity on cos^2^ θ. (**c**) The dependence of PL peak position on cos^2^ θ.

In our results, the PL intensity of monolayer MoS_2_ with silver nano-antennas is significantly enhanced compared to the bare monolayer (Fig. 2a). The maximum enhancement is approaching 10 times when the incident polarization is of θ = 0°. Interestingly, the enhancement shows an obvious polarization angle dependence: the enhancement monotonically decreases when θ increases from 0°–90°. To better understand the polarization-dependent PL intensity enhancement, we draw the integrated PL peak intensity vs cos^2^ θ (Fig. 2b) and obtain an nonlinear relation with offset of about 4 at cos^2^ θ = 1. We also examined the PL peak position (Fig. 2c) with polarization angle θ. It is obvious that the PL peak position displays a monotonic dependence on the θ. The PL peak red-shifts about 20 meV when cos^2^ θ increases from 0 to 1 and shows saturated behavior. This result shows that the incident polarization can not only tune the PL peak intensity but also the peak position.

## Discussions

**Figure 3:**
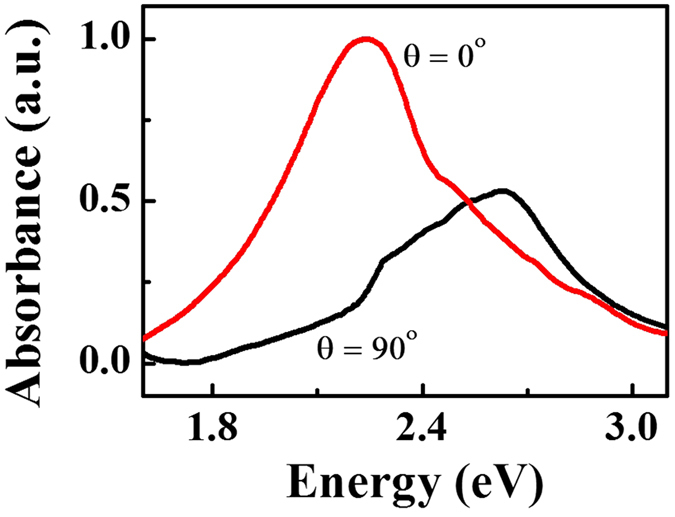
The measured absorption of Ag nano-antenna in our experiment. The peak wavelength of transverse and longitudinal mode are at 2.64 eV (470 nm) and 2.24 eV (555 nm), respectively. The extinction ratio at 2.34 eV (532 nm) of longitudinal mode to transverse mode is about 3, giving rise to the offset of PL in the case of θ = 90°.

From our design, only the longitudinal mode has resonance with the excitation laser and the transverse mode has negligible effect. So when our polarized excitation is with θ angle to the long axis of nano-antennas, the effective electric field will have a scaling factor of cosθ. Intuitively, the PL integrated intensity should be proportional to the electric field squared and show a quadratic dependence on cos θ, i.e. the enhancement should have a linear relation with cos^2^ θ without any offset. The observed offset is attributed to the non-perfect shape of nano-antenna, where the transverse mode contributes about 30% absorption (Fig. 3). The nonlinear shape in Fig. 2b is believed to relate to the PL profile evolution and will be discussed in the latter part.

Several mechanisms may explain the polarization dependent PL peak position evolution: 2H-1T phase transition^27^, thermal effect^28^, and many-body effects^29^,^30^.

**Figure 4:**
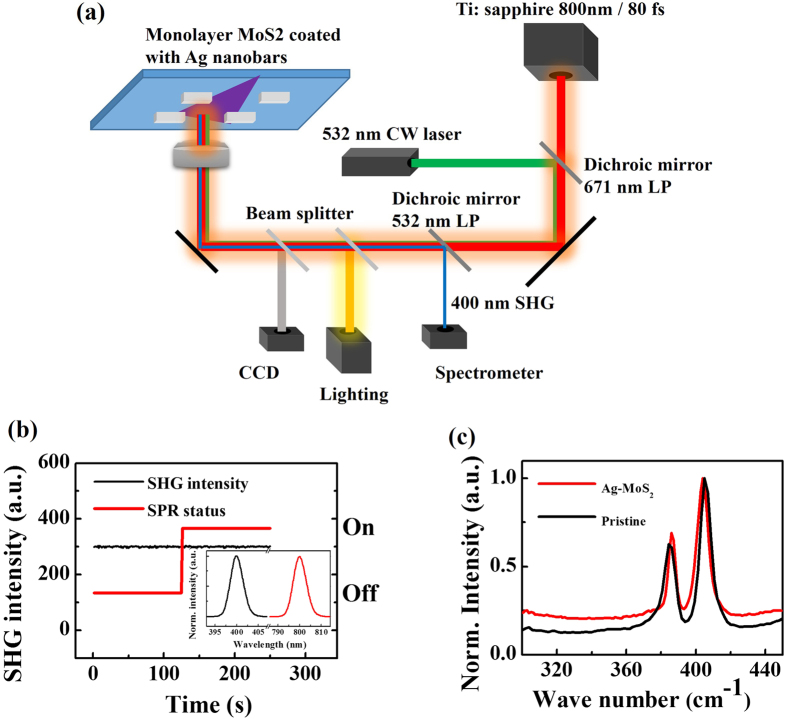
Phase transition test. (**a**) Schematic diagram of the experimental setup for testing phase transition of MoS_2_ monolayers. Another continuous 532 nm semiconductor laser was added to excite surface plasmon resonance. The Ag nano-antenna structure arrays were designed in size of 40 × 40 × 80 nm for single structure. (**b**) SHG intensity variations of Ag antennas coated monolayer MoS_2_, with and without additional 532 nm laser to excite SPR. Inset: the spectra of the SHG signal (black) from the monolayer MoS_2_ and from the incident femtosecond 800 nm laser (red). (**c**) Raman spectrum of Ag nano-antenna coated monolayer MoS_2_.

For 2H-1T phase transition, the crystalline structure will change from non-centrosymmetric to centrosymmetric. Essentially, second-harmonic-generation (SHG) signal is very sensitive to the crystalline symmetry of a materials^31^,^32^,^33^. As a result, SHG generation will be strong and negligible for non-centrosymmetric 2H monolayer MoS_2_ phase (ordinary phase) and centrosymmetric 1T phase (possible phase induced by light excitation), respectively. We carried out SHG experiment (800 nm 100 fs) with and without SPR excitation (Fig. 4a), and the SHG signal doesn’t disappear under light excitation (Fig. 4b). Moreover, we didn’t clearly observe the three characteristic 1T Raman peaks^27^ in Fig. 4c. All these results above implied that the crystal structure and the original asymmetry were not changed. Therefore, the 2H-1T phase transition could be excluded in our experiment.

For thermal effect, the observed PL shifts of nearly 0.02 eV in experiments would be caused when temperature was heated by ~ 100 K^28^, while temperature rise by SPR was estimated to be ~ 10 K^27^ under the current illumination intensity in our measurements. Hence, the thermal effect is neither the dominant factor for PL shifts.

SPR-excited-hot-electrons^34^,^35^,^36^ with energy higher than the Schottky barrier between metal structure and MoS_2_ can skip from metal into MoS_2_ and dope it effectively. The optical response in two-dimensional MoS_2_ are excitonic in nature and PL peak of intrinsic MoS_2_ are related to the lowest energy 1 s exciton. However, when hot electron injects into the MoS_2_, it effectively dopes the MoS_2_ and will lead to a new quasi-particle states of trion (or charged exciton). The trion effects were first observed in electrically gated MoS_2_^29^.

**Figure 5:**
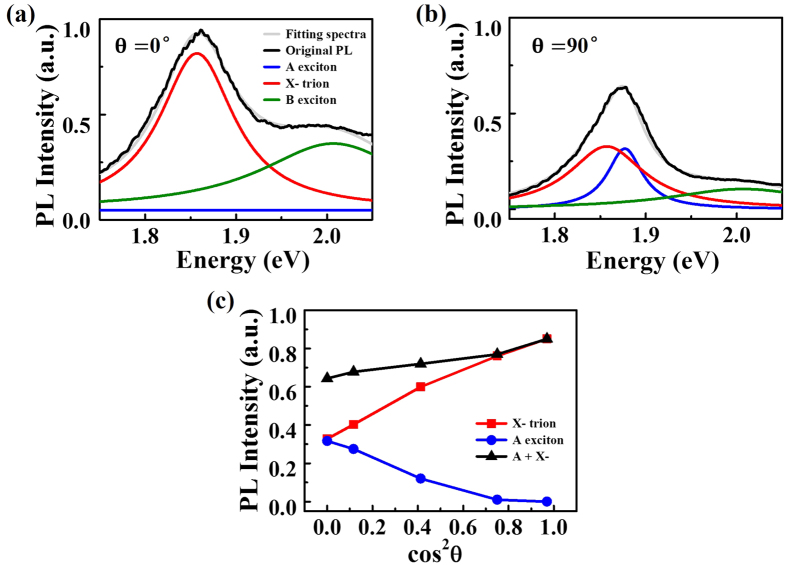
The evolution of trion and exciton under different θ (a,b). The decomposed PL intensity into trions and excitons under 0^o^ and 90^o^ incident polarization, respectively. (**c**) Integrated PL intenstiy evolution of trions and excitons with cos^2^ θ.

To obtain more quantitative information of the many-body trion effects, we decompose the PL spectra into A, B excitons and trions X- under different θ (Fig. 5a,b) by employing Lorentzian fitting, following the method in refs 29,37. The optimal fitting center energy of A, B excitons and trions X- are 1.883 eV, 2.007 eV and 1.857 eV, respectively. The fitting FWHM of A, B excitons and trions X- are 0.0548 eV. 0.2120 eV and 0.0918 eV, respectively. It is observed that, under cos^2^ θ = 0, or the smallest field enhancement, exciton accounts for the largest proportion in the PL peak. However, when cos^2^ θ increases (Fig. 5c), the trion portion increases gradually and finally reach its maximum at cos^2^ θ = 1, while exciton portion decreases to zero. Since the trion energy was about 20 meV, a number lower than that of the exciton energy, the combined peak position redshifts will show saturation behavior, agreeing well with our experimental observation in Fig. 2c.

These exciton-trion competition mechanism is also the origin for the nonlinear shape in Fig. 2b. When cos^2^ θ increases, although the longitudinal field enhancement will contribute an increased weighting factor of cos^2^ θ in PL intensity, the hot carrier doping becomes stronger at the same time. The hot carrier induced trion will result in a decreasing of the PL intensity simultaneously. These two opposite effects compete with each other and the net effect is that the PL enhancement is monotonically increasing with cos^2^ θ, but smaller than the predicted linear relation by pure field calculation.

In summary, we have demonstrated an enhanced photo-response in MoS_2_ by utilizing surface plasmonic resonance (SPR) based on metallic nano-antenna with lateral characteristic size of 40 × 80 nm. The enhanced photo-response is realized by the matching of resonance of nano-antanna with our incident laser energy. The photo-response shows obvious polarization dependence: the PL intensity will be enhanced from 10 to 4 times, and the PL peak position will blueshift 20 meV when polarization changes from being parallel to vertical to the long axis of the nano-antenna. This polarization dependent behavior originates from angle dependent field enhancement along long axis of the nano-antenna and is associated with exction-to-trion evolution. Our method should be versatile to enhance and control the optical response of other 2D materials, such as MX_2_, and therefore be useful for the coming optoelectronic applications of 2D materials.

## Methods

### Sample preparation

Monolayer MoS_2_ samples, typically 30 um^2^, were controllably synthesized on mica through a low-pressure chemical vapor deposition (LPCVD) process and then transferred onto SiO_2_ (300 nm)/Si substrate. The sample thickness of monolayer was confirmed by photoluminescence measurements and atomic force microscopy (NTEGRA SNOM, NT-MDT), respectively. Ag nano-antenna arrays were fabricated through a traditional electric beam lithography (e-LINE plus, RAITH) method and then followed by laser molecular beam epitaxy (LMBE450, KYKY).

### Optical measurements

The photoluminescence measurements were performed using a single mode semiconductor laser centered at 532 nm. The laser was kept at 4.5 mW power and focused onto monolayer MoS_2_ with and without Ag nano-antenna (40 × 80 × 35 nm) arrays by a 60 × objective (NA = 0.85, Edmond). The SHG experiment (phase transition test) was performed by using an 800 nm femtosecond laser (80 fs, 82 MHz, Tsunami) focused onto monolayer MoS_2_ coated with Ag nano-antenna arrays by a 60 × objective (NA = 0.75, Edmond). The power of 800 nm femtosecond laser was kept at 5 mW. The set-ups of SHG experiment were elaborated in the supporting material. The PL and SHG signals were collected and analyzed with a grating spectrometer (SP2500i, PIActon) equipped with a liquid nitrogen cooled CCD. The Raman spectrum was measured by UV Raman Spectrograph (invia 6365, Renishaw).

### Numerical Simulation

The absorption spectra of Ag nano-antenna arrays were simulated by finite element method using Comsol Multiphysics 4.3b software.

## Additional Information

**How to cite this article**: Li, J. *et al.* Tuning the photo-response in monolayer MoS_2_ by plasmonic nano-antenna. *Sci. Rep.* **6**, 23626; doi: 10.1038/srep23626 (2016).
